# Ocular Pentastomiasis in the Democratic Republic of the Congo

**DOI:** 10.1371/journal.pntd.0003041

**Published:** 2014-07-24

**Authors:** Mihály Sulyok, Lajos Rózsa, Imre Bodó, Dennis Tappe, Richard Hardi

**Affiliations:** 1 St. István & St. László Hospital, Dept. of Infectious and Tropical Diseases, Budapest, Hungary; 2 MTA-ELTE-MTM Ecology Research Group, Budapest, Hungary; 3 University of Debrecen, Department of Evolutionary Zoology and Human Biology, Debrecen, Hungary; 4 St. István & St. László Hospital, Department of Hematology and Stem Cell Transplantation, Budapest, Hungary; 5 National Reference Center for Tropical Diseases, Bernhard Nocht Institute for Tropical Medicine, Hamburg, Germany; 6 St. Raphael Ophthalmological Center, Ophthalmological Ambulance, Mbuji Mayi, Democratic Republic of Congo; University of Melbourne, Australia

## Abstract

Ocular pentastomiasis is a rare infection caused by the larval stage of pentastomids, an unusual group of crustacean-related parasites. Zoonotic pentastomids have a distinct geographical distribution and utilize reptiles or canids as final hosts. Recently, an increasing number of human abdominal infections have been reported in Africa, where pentastomiasis is an emerging, though severely neglected, tropical disease. Here we describe four ocular infections caused by pentastomids from the Democratic Republic of the Congo. Two cases underwent surgery and an *Armillifer grandis* infection was detected by morphological and molecular approaches. Thus far, 15 other cases of ocular pentastomiasis have been reported worldwide. Twelve cases were caused by *Armillifer* sp., recorded almost exclusively in Africa, where such infections occur as a consequence of hunting and consuming snakes, their final hosts. Seven further cases were caused by *Linguatula serrata*, a cosmopolitan pentastomid whose final hosts are usually canids. Intraocular infections caused permanent visual damage in 69% and a total loss of vision in 31% of reported cases. In contrast, ocular adnexal cases had a benign clinical course. Further research is required to estimate the burden, therapeutic options and pathogenesis of this neglected disease.

## Author Summary

Ocular pentastomiasis is a severely neglected parasitic disease. Most human infections are linked to the consumption and/or handling of snakes. Here we present a series of cases from a remote area of the Democratic Republic of the Congo. Two patients had an infection with *Armillifer grandis*. As livestock production in Congo Basin countries is limited, people consume “bushmeat” from wild animals, including snakes. On the other hand, developed countries increasingly import reptiles from tropical countries as pet animals, which represents a potential threat to the public. Fifteen human cases of ocular pentastomiasis have been described in the literature; however, this disease may be under-reported in rural areas of the tropics. Here, we review human cases as well as key aspects of the epidemiology, clinical features, treatment and prevention of this forgotten disease.

## Introduction

Pentastomiasis is a neglected zoonotic disease caused by the larval stage (nymphs) of pentastomids, a unique and enigmatic group of crustacean-related parasites. The parasites usually have an indirect lifecycle, involving various intermediate and definitive hosts. *Linguatula serrata*, a species occurring in temperate climatic regions of the world, utilizes canids as definitive hosts, whereas *Porocephalus* species in America and *Armillifer* species in Africa and Asia (*Armillifer armillatus* in West Africa, *A. grandis* in Central Africa, *A. agkistrodontis* and *A. moniliformis* in Asia) inhabit snakes as final hosts [1]. In the respiratory tract of the final host, the adults produce a large number of infective eggs, which are excreted via respiratory and enteral secretions. The eggs then infect suitable intermediate hosts (often rodents and small non-human primates in the case of *Armillifer* infection). Humans can become accidental intermediate (dead-end) hosts. After ingestion of infective ova, the nymphs hatch in the gut of the intermediate host and invade the viscera, where they grow and moult several times to become infective. Transmission to definitive hosts occurs when an infected intermediate host falls prey to a suitable predator. The nymphs then migrate to the respiratory tract of the predator, where they attach to the mucosa with two pairs of circumoral chitinous hooklets, develop into adults and then reproduce sexually [2].

In humans, pentastomid larvae typically invade the peritoneum, liver, spleen, mesentery and pleura, causing visceral pentastomiasis [1]. Infection is usually asymptomatic [1]; however, symptomatic [3], severe [4] and even fatal [5] *Armillifer* infections have also been reported. Risk factors of this infection include the handling of snakes or snake products, consumption of undercooked snake meat, and possibly snake farming and snake totemism [1], [6], [7]. *Armillifer armillatus* is the second most encountered pentastomid species in humans after *L. serrata*, with the majority of cases reported from Ghana and the Congo region [8]. Disease due to *A. grandis* is rare [1], [8], the first case having been described in 1966 in the Congo Basin [9]. Ocular pentastomiasis is a rare manifestation. Here, we present four severe cases from the Democratic Republic of the Congo (DRC) detected by classical and/or molecular diagnostic methods. We also review all previously published ocular infections and discuss the epidemiology, clinical features, treatment and prevention of this neglected tropical disease.

## Materials and Methods

### Ethics statement

The Ethics Committee of the St. Raphael Ophthalmological Center in Mbuji Mayi approved the present study. All adult subjects and the parents of child participants provided informed consent. Oral informed consent was obtained due to illiteracy and was documented in the outpatient files. The Ethics Committee approved the use of oral consent.

### Case series

From 2008 to 2012, we examined approximately 4000 patients with eyesight problems during our ophthalmology missions to the Sankuru district, in the vicinity of Kole, DRC. Overall, we identified four patients with ocular pentastomiasis and associated eye damage. The calculated prevalence was, thus, 0.001 among inhabitants with ocular problems.

### Case 1

**Figure 1:**
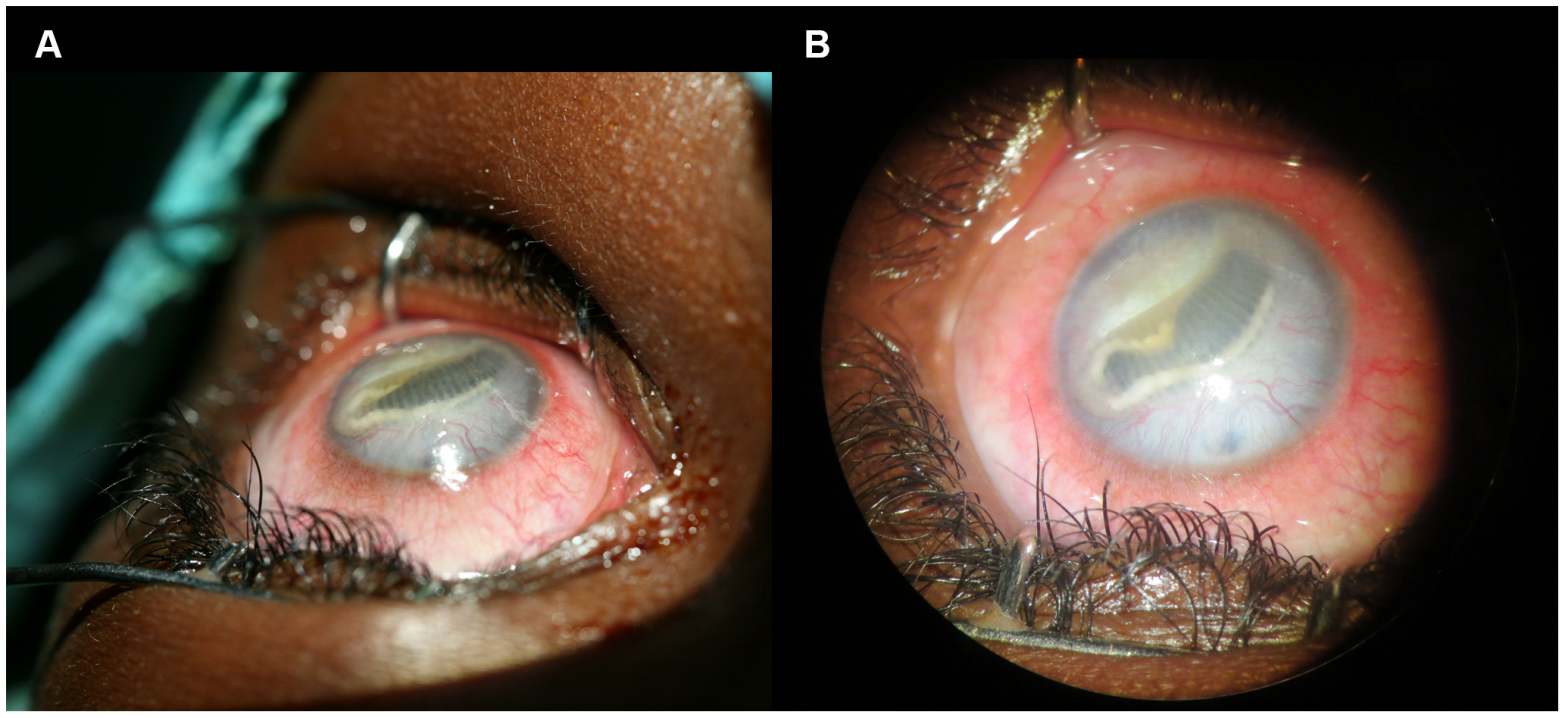
Annulated foreign body in the anterior chamber of the left eye from Case 1. **A**, lateral view. The eye shows marked conjunctival injections and the foreign body fills the whole pupil blocking the eyesight completely. **B**, frontal view. The high number of annulations of the parasite is clearly visible.

**Figure 2:**
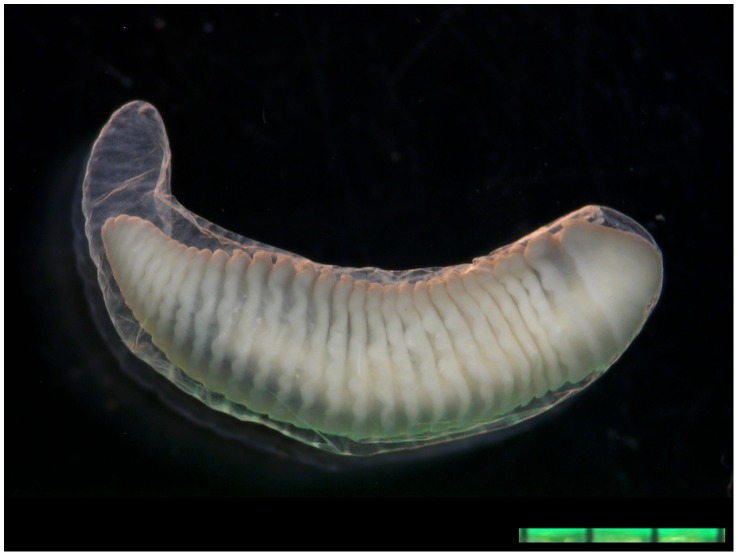
Extracted pentastomid nymph from Case 1, morphologically and molecularly identified as *Armillifer grandis*. The nymph is still surrounded by its shed translucent cuticle (exuvia), a characteristic feature of pentastomid larvae as they moult in the intermediate hosts' tissues. *A. grandis* is the smallest of the zoonotic African *Armillifer* species with the most body annulations (9–15 mm body length and >25 annulations; *A. armillatus*, the nearest geographical neighbour for comparison: 12–23 mm length and 18–22 annulations (2). *A. grandis* is the least often encountered zoonotic *Armillifer* species worldwide. Scale bar = 3 mm.

An 11-year-old girl was referred to our outpatient ophthalmology mission, an annual two-week mobile clinic in Kole. The girl had been complaining of pain, redness and decreased vision in the left eye for four months. The visual acuity was severely impaired, with light perception only in all four quadrants of the left eye, while remaining 10/10 in the right eye. On examination, her right eye appeared normal. The left eye showed mild ciliary and conjunctival injection. The cornea was transparent, with some neovascularization. An annulated foreign body was identified in the anterior chamber with peristaltic motion (Figure 1) consistent in morphology with a pentastomatid. The iris was covered by a fibrinous membrane, which also obstructed the pupil, rendering the rest of the eye unsuitable for examination. The eye was markedly hypotensive. The eye was clipped under retrobulbar anesthesia, and the cornea was incised at the limbus with a 15° knife; the parasite was extracted from the anterior chamber (Appendix Video). The parasite was 10 mm long and 2 mm wide, with 31 clearly visible annulations. The organism was surrounded by a transparent capsular-like cuticle, and showed intense peristaltic movements after removal. Two pairs of hooklets were present on each side of the mouthpart, and the parasite was identified morphologically as a larval stage of *Armillifer grandis* (Figure 2). Using an 18S rRNA gene marker, a pentastomid-specific PCR [6] was performed on genomic DNA derived from the excised parasite specimen. The resultant amplicon of 377 bp was sequenced and deposited in the GenBank database (accession no. KM023155). The sequence had 99% similarity to those representing *Armillifer armillatus* (GenBank accession no. HM756289; query coverage 94%, 0 gaps), *A. agkistrodontis* (accession no. FJ607339; query coverage 100%, 1 gap) and *A. moniliformis* (accession no. HM04870; query coverage 100%, 1 gap). The present specimen was unequivocally identified as an *A. grandis* nymph based on size and number of annulations [2]; there was no sequence for *A. grandis* in any current database. Unfortunately, the patient lost vision in the left eye, despite surgery. This patient reported handling snakes regularly and suffered an eye-splash accident with body fluids from a snake during food preparation approximately six months prior to presentation.

### Case 2

A 36-year-old male patient presented to one of our ophthalmology missions in Lokoko, from a village in the Pelenge area, DRC. He has been suffering from visual disturbances in the left eye for 3 years. Symptoms had begun with redness and pain. The pain stopped some time ago, but the visual problem persisted. The right eye was normal with intact vision. Vision in the left eye was severely impaired with light perception only. The conjunctiva, cornea, lens and the anterior chamber were without any detectable abnormalities, but an approximately 15 mm long and 5–6 mm wide parasite reminiscent of a pentastomid nymph was floating freely in the vitreous body, directly behind the lens, encapsulated in a translucent cyst. The annulations could not be precisely counted, but were estimated to be over 20. There was a remarkable absence of any sign of inflammation in the eye. The retina was detached approximately 270 degrees, with the detached part floating freely. The patient declined surgical intervention and was reexamined one year later. At that time, the retina had completely detached (360 degrees), and the patient lost all light perception in the affected eye. Surgery was again offered, but the patient did not consent. He could not recall any trauma, but admitted to consume snake meat regularly.

### Case 3

A 63-year-old woman from the Pelenge region presented to the same mission as case 2. She had lost vision in the right eye 3 years prior to presentation. The left eye was assessed as normal during slit lamp and fundus examination. In the right eye, there was an annulated, crescent-shaped parasite in a subretinal localization, positioned nasally from the papilla. There were no signs of retinal detachment. The parasite showed no movement, even upon stimulation by pressing on the eye, and, thus, appeared to be dead. The parasite was ∼8 mm long, 1.5 mm wide and had >20 annulations. Based on these findings, the diagnosis of ocular pentastomiasis caused by an unidentified species was made. Due to the localization of the parasite, surgery was not attempted. The patient consumed snake meat regularly.

### Case 4

A 25-year-old male presented with blindness and pain in his left eye. Ophthalmologic examination showed a shrunken, non-functional eye. The pupil was nonreactive to light. Using a slit lamp, a vermiform foreign body was seen in the anterior chamber. The parasite was motile and was removed under local anesthesia. This case was previously reported as a photo quiz [10]. Subsequently, the extracted nymph was morphologically and molecularly examined (unpublished data). The parasite was 9 mm long and 2 mm wide, had 30 annulations, and was surrounded by a partially shed transparent cuticle. The 18S rRNA sequence was the same as that isolated from case 1. Thus, this case was caused by *A. grandis* (unpublished data). This patient also regularly consumed snake meat.

### Methods of literature review

An electronic literature search was conducted using PubMed (MEDLINE). The following Medical Subject Heading terms were used: Pentastomida; Eye infections, Parasitic; Eye/Parasitology. The full texts of the articles selected were reviewed by all authors. The references in all publications were also reviewed to identify additional articles that did not appear in the initial search. Articles in German, French, and Portuguese were also included.

## Results and Discussion

### Epidemiology

**Figure 3:**
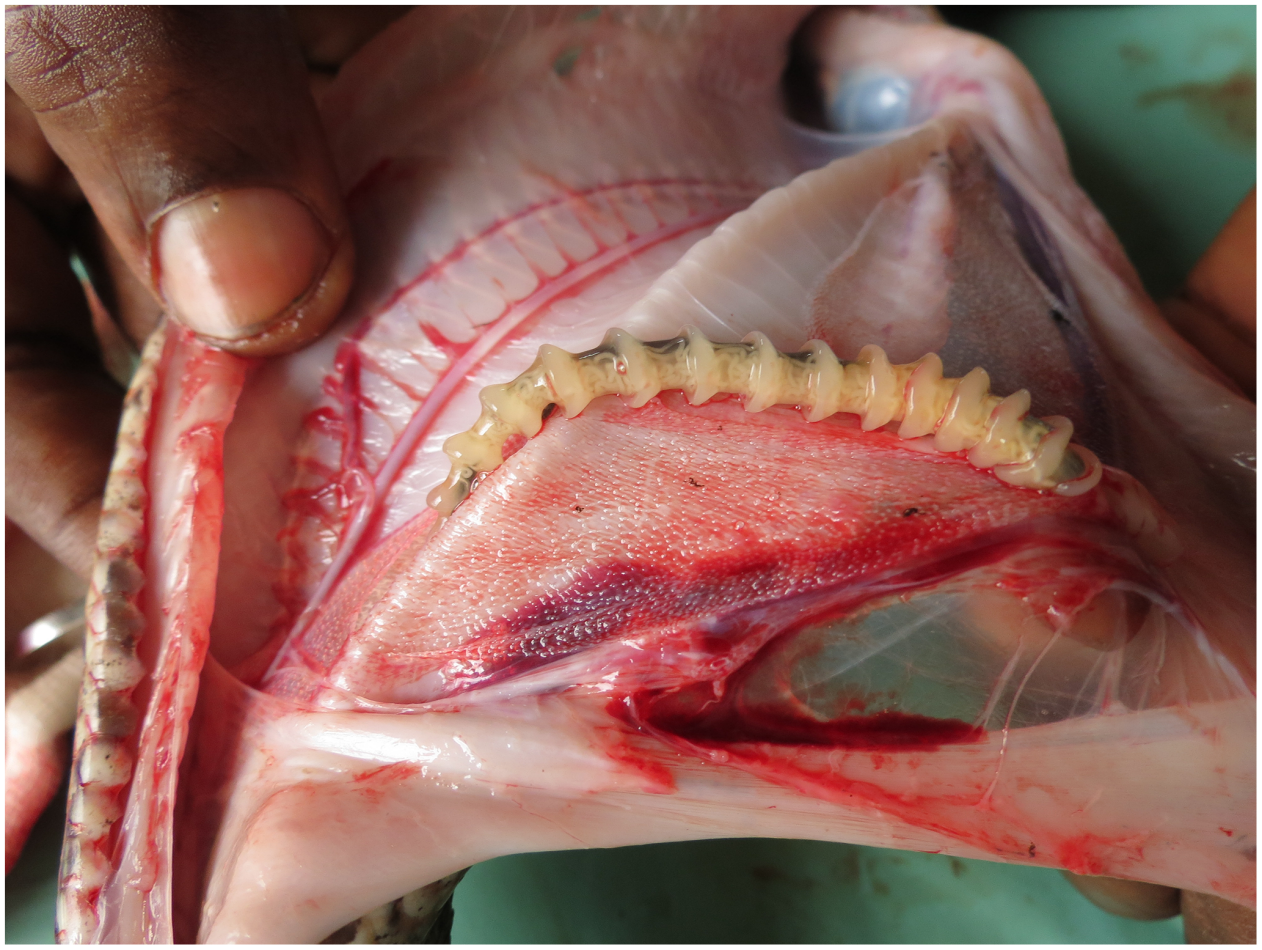
Local villagers often find pentastomids in snakes sliced for consumption. Adult *Armillifer armillatus* (confirmed by 18S rRNA gene PCR) in the lung of a young Ball Python (*Python regius*) before consumption in Sankuru district, Democratic Republic of the Congo. Sometimes local people do not clean and cook the snake properly just spit out the parasites while eating, so the eggs may accidentally be swallowed. The ova may also contaminate snake meat during butchering. Unless properly cooked, an egg may hatch in the intestine of an accidental human intermediate host. While most larvae migrate to visceral organs, a few will make their way to the eye [1].

The true number of patients affected by pentastomiasis is unknown, even estimates are lacking. This can be explained by the fact that visceral pentastomiasis is often asymptomatic [1]. However, this disease might be more prevalent than expected in some parts of the world, as autopsy studies in Nigeria and West Malaysia have shown prevalences up to 33–45% in some populations [11], [12]. Ocular pentastomiasis, though a rare form of the disease, is likely to be detected more readily than the visceral manifestation, because an eye infection produces overt symptoms. The 0.001 prevalence among patients with vision problems is a clear indication that pentastomiasis is relatively prevalent in this geographic region where inhabitants frequently consume snakes. Thus, it is likely that ocular pentastomiasis represents only the ‘tip of the iceberg’ of all pentastomiasis forms. Ocular pentastomiasis may thus be regarded as a sentinel form of all forms of pentastomiasis that might otherwise remain undetected. In this region of the DRC, local villagers often find adult pentastomids in the snakes they consume (Figure 3). Snakes can also be eaten ritually as part of the ju ju rituals in Africa (e.g., Benin, Nigeria, Cote d' Ivoire, Cameroon, DRC), or Malaysia (Temuan tribe) [13]. Epidemiological risk factors and possible routes of transmission were not determined in previously published reports of ocular pentastomiasis. The consumption of poorly cooked snake meat had occurred regularly in all of our four cases. In case 1, an eye-splashing accident with body fluid from a snake occurred two months prior to the onset of clinical symptoms. No similar accidents had been reported in any other published cases. However, this patient also consumed snakes regularly, so the possible direct transmission to the eye of *A. grandis* nymphs remains speculative. Interestingly, in case of *Linguatula* eye infections, ocular trauma was described in two cases prior to the onset of symptoms [14], [15]. In one case, a fly had hit the eye, and in the other a ball. Three *Linguatula* patients had kept pet dogs [16], [17], [18]. These circumstances may all be coincidental, but in theory, pentastomid eggs might be mechanically transmitted to the conjunctiva, although such direct transmission has yet to be proven.

Besides our 4 patients described here, 15 other cases of ocular pentastomiasis have been reported in the literature (Table 1 and Table 2). Among these 19 cases, 12 were male, 6 were female and in one case gender was not reported. The median age at diagnosis was 14 years. Most cases (11/19) were reported from sub-Saharan Africa or found in patients originating from this region. The remaining cases were reported from the United States, Europe, India, Israel and South America. All patients from Africa had *Armillifer* infections, most often relating to *A. armillatus*. However, some degree of uncertainty surrounds the specific detection of *A. armillatus*, which closely resembles *A. grandis*. To this point, we observe that in all but one previous case reports, the parasites were identified by morphological examination only (the single exception is a *Linguatula* infection verified by PCR [18]). In two of our cases, the larvae were not removed, so that the only diagnostic clue was through fundoscopic examination. However, our other two cases represent the first published unequivocally proven intraocular infections by *A. grandis*. There is a case of *A. grandis* infestation extraocularly in the eyelid of a patient from the neighboring region, Kisangani District, Zaire but diagnosis was based exclusively on morphological features of the parasite [19]. Cases of ocular pentastomiasis from outside Africa have been almost exclusively caused by *L. serrata*. Only one patient was described to have an infection with *Porocephalus* sp. Given that this particular patient had formerly visited both continents, the parasite in his eye could either be a South-American *Porocephalus* or an African *Armillifer* species [20].

**Table 1:** Epidemiological and parasitological characteristics of patients with ocular pentastomiasis reported in the literature, including current cases.

Case Nr.[Reference]	Year*	Country	Age/sex	Diagnosis	Pentastomid Species
Case 1 [14]	1962	USA	8/F	Morphology	*L. serrata*
Case 2 [15]	1987	Israel	12/M	Morphology	*L. serrata*
Case 3 [16]	2011	India	5/M	Morphology	*L. serrata*
Case 4 [17]	1960	USA	16/M	Morphology	*L. serrata*
Case 5 [18]	2011	Austria	14/F	PCR	*L. serrata*
Case 6 [21]	1951	Congo†	10/M	Morphology	*A. armillatus*
Case 7 [24]	1967	Liberia	50/M	Morphology	*A. armillatus*
Case 8 [29]	1962	Liberia	9/M	Morphology	*A. armillatus*
Case 9 [22]	1957	Liberia	6/M	Morphology	*A. armillatus*
Case 10 [23]	1962	Ghana‡	25/M	Morphology	*A. armillatus*
Case 11 [23]	1962	Uganda‡	4/F	Morphology	*A. armillatus*
Case 12 [31]	1979	Ghana	15/M	Morphology	*A. armillatus*
Case 13 [20]	1972	Brazil	38/M	Morphology	*Porocephalus* sp.
Case 14 [25]	1964	Portugal	9/ND	Morphology	*L. serrata*
Case 15 [26]	1999	Ecuador	34/F	Morphology	*L. serrata*
Current Case 1	2014	DRC	11/F	PCR	*A. grandis*
Current Case 2	2014	DRC	36/M	Morphology	Unidentified pentastomid
Current Case 3	2014	DRC	63/F	Morphology	Unidentified pentastomid
Current Case 4 [10]	2013	DRC	25/M	Morphology	*A. grandis*.§

DRC, Democratic Republic of the Congo; ND, not described in publication;

*, year of publication;

† formerly Belgian Congo;

‡ patients originated from that country;

§ this case has currently been re-analyzed morphologically and by PCR by our group and is identified as *A. grandis*.

**Table 2:** Clinical characteristics of patients with ocular pentastomiasis, including the current cases.

Case Nr. [Reference]	Relevant history	Side	Localization	Symptom duration	Motile parasite	Therapy	Eye sight
Case 1 [14]	Ocular trauma†	Right	AC	4 days	Yes	Surgical	Severely damaged
Case 2 [15]	Ocular trauma‡	Right	AC	1 week	Yes	Surgical	Severely damaged
Case 3 [16]	Pet dog	Right	AC	2 months	No	Surgical*	Regained after surgery
Case 4 [17]	Pet dog	Right	AC	2–3 months	Yes	Surgical	Intact
Case 5 [18]	Pet dog, cat, turtle	Right	AC	ND	Yes	Surgical	Regained after surgery
Case 6 [21]	ND	Right	SC	ND	Yes	Surgical	Intact
Case 7 [24]	Eating snakes	Right	AC	6 months	No	Surgical	Severely damaged
Case 8 [29]	ND	Left	Eyelid	12 months	ND	Surgical	Intact
Case 9 [22]	ND	Left	SC	Few weeks	ND	Surgical	Intact
Case 10 [23]	ND	Right	PC	Several weeks	No	None	Severely damaged
Case 11 [23]	ND	Left	SC	4 months	Yes	Surgical	Intact
Case 12 [31]	ND	Left	LC	ND	ND	Surgical	Intact
Case 13 [20]	Eating snakes	Left	SC	6 weeks	Yes	Surgical	Intact
Case 14 [25]	ND	Left	PC	6 months	Yes	Surgical	Severely damaged
Case 15 [26]	None	Right	AC	2 months	Yes	Surgical	ND
Current Case 1	Eating snakes§	Left	AC	4 months	Yes	Surgical	Severely damaged
Current Case 2	Eating snakes	Left	PC	36 months	No	None	Severely damaged
Current Case 3	Eating snakes	Right	PC	36 months	No	None	Severely damaged
Current Case 4 [10]	Eating snakes	Left	AC	ND	Yes	Surgical	Severely damaged

ND, not described in publication; AC, anterior chamber; PC, posterior chamber; LC, lacrimal caruncule; SC, subconjunctival;

*, before surgery steroids and albendazole were administered;

† ball hit his eye before symptom onset;

‡ a fly hit his eye before symptom onset;

§ also had an eye-splash accident with the body fluids of a snake six months before symptom onset.

### Clinical findings

The time from the onset of symptoms to diagnosis (where reported; 15 cases) varied from 4 days to 36 months. The parasite resided in the ocular adnexa in six cases (4 subconjunctival, 1 nasolacrimal and 1 eyelid infestation) and was found intraocularly in the remaining 13 cases. In nine patients, the pentastomid nymphs were located in the anterior and in four patients in the posterior chamber (Table 1.). Given the observed motility of the parasite, pentastomid larvae can possibly change their location within the eyeball. A nymph was observed to escape from the anterior to the posterior chamber during an attempt of extraction [18].

#### 1. Ocular adnexal localization (Table 1)

Two patients with subconjunctival infection were asymptomatic [21], [22]. In case 10, the patient had been suffering from periorbital edema for 4 months [23], while in case 13, a subconjunctival mass had been growing also causing conjunctival injection for six weeks [20]. The nymphs were typically located within their cyst-like translucent shed cuticle (exuvia). In one of these patients (case 6), two separate cysts were present containing one parasite each [21]. The nymphs were removed in all cases. No visual loss or any permanent damage has been reported in patients with adnexal localization. All African parasites were morphologically identified as *A. armillatus*, and there was one questionable case (case 13) of infection with *Porocephalus* sp. from South America. Signs of extraophthalmical infestation were not searched for in any of the patients.

#### 2. Intraocular manifestation

In 13 patients with intraocular infection (including the current cases) signs and symptoms were the following: decreased vision (12/13 patients, 92%), eye pain in 8/13 (62%), conjunctival injection in 5/13 (39%), and photophobia, excessive lacrimation in 1/13 (7.7%) [10], [14]–[18], [23], [24], [25], [26]. In cases of posterior chamber localization, retinal detachment occurred in 3/4 cases (75%). Iridodonesis, lens subluxation and floating of the nymph behind the lens occurred in 1/4 cases (25%) [23], [25]. Anterior chamber localization caused iritis/uveitis in 5/9 patients (56%) with or without elevated intraocular pressure and goniosynechiae. Iridodonesis was present in 3/9 (33%), dislocated lens in 2/9 patients (22%) [10], [14]–[18], [24], [26]. Intraocular parasites caused permanent visual damage in 9/13 (69%) and total loss of vision in 4/13 cases (31%). *L. serrata* was the causative agent in seven, *Armillifer* sp., not further specified, in three, *A. grandis* in two cases, and *A. armillatus* in one case (Table 1.). Identification was based on morphology of the nymphs in 10/13 and on PCR in 3/13 patients. The parasites were removed successfully in 10/13 of the cases [10], [14]–[18], [23], [24], [25], [26]. Surgical intervention could improve vision in two patients only (Table 2.)

### Therapy

There are no published studies assessing antiparasitic treatment of human pentastomiasis; however, the use of ivermectin, praziquantel and mebendazole have been suggested [13]. In cases with ocular localization of the parasites, surgical removal is the treatment of choice. However, in rural areas, where most cases of pentastomiasis occur, medical and surgical services are often unavailable. Parasites were surgically removed in 15 cases (Table 2.), while in four patients, no intervention or medical therapy was attempted. The optimal timing of surgical extraction is unknown. Considering that the nymphs are viable for approximately two years in the human body [1], and that dying and antigen-releasing parasites may provoke stronger host immune responses [1], removal as early as possible seems to be advisable. An early extraction will not only improve the quality of life rapidly but also will prevent further organ damage, as living nymphs are motile and feed on components of the eyeball. Ingested hemoglobin found in parasites from our cases 1 and 4 suggest that pentastomids cause direct damage to intraocular structures. In cases of intraocular localization, nymphs were removed through a corneoscleral/limbal incision. Vitrectomy, iridectomy and lens removal were also performed in two cases of posterior chamber localization.

### Prevention

The prevention of pentastomiasis should focus on personal hygiene measures when handling snakes and snake products, such as proper hand washing after snake contact. The consumption of undercooked reptile meat and organs should be avoided. Since livestock production in the Congo Basin rainforests is limited, inhabitants rely on other sources of protein for their diet; consequently, “bushmeat” consumption plays an important role in this region. As populations of the most desired mammals are being increasingly exploited, people in rural areas predictably turn to consuming more reptiles [27]. On the other hand, developed countries increasingly import reptiles from tropical countries as pets, which represents a potential threat to the public. It should be kept in mind that pet owners, as well as veterinarians, zoo and snake farm workers can be infected via respiratory secretions or faeces. Nymphal linguatuliasis can be prevented by hand washing after contact with dogs or their excreta/secreta [28].

### Conclusions

Pentastomiasis is usually an asymptomatic infection. However, when pentastomid larvae occur in the eye, the consequences can be devastating. Our case series suggests that in central DRC, this disease is more common than previously thought. Case reports from Liberia [22], [24], [29] indicate a similar situation. Here we conclude from a review of all reported cases in the medical literature, and our own experience, that extraocular localization of pentastomid nymphs has good prognosis after surgical treatment, while intraocular parasites usually cause permanent visual damage, despite surgical intervention. The early removal of intraocular nymphs in a well-equipped medical center seems to be crucial to conserve sight [18], while cases in rural sub-Saharan regions with no available medical services have a poor prognosis. Interestingly, *A. grandis*, an otherwise very rarely encountered pentastomid species, was responsible for at least two of the four cases described here. Although the pathogenesis of ocular pentastomiasis is unknown, pentastomid nymphs likely reach the eye via the bloodstream, similar to cases of neurocysticercosis after the oral ingestion of infective *Taenia solium* eggs [30]. Direct contamination, although unlikely, cannot been ruled out, particularly in cases involving the ocular adnexa after traumatic injuries. Preventive measures should include the proper cooking of snake meat before consumption and hand washing after handling snakes. Further epidemiological studies in this region of relatively high prevalence are required to estimate disease burden, study the pathogenesis and evaluate therapeutical options of this seriously neglected disease.

**Key Learning Points**

- Ocular pentastomiasis is caused by the nymphs (larval stages) of the tongue worms *Linguatula serrata*, *Armillifer armillatus*, and *Armillifer grandis*.
- There is insufficient data regarding epidemiology, treatment, and prevention of ocular pentastomiasis, a severely neglected disease.
- Ocular pentastomiasis due to *Armillifer* species is linked to handling or consuming infected snakes. Livestock production in the Congo basin is limited, and the local human population relies on other sources of protein for their diet. As a consequence, “bushmeat” consumption plays an important role in this region, excessively exploiting mammal species, leading also to the consumption of reptiles.
- The reported cases of ocular infection presumably represent the ‘tip of the iceberg’ only, indicating that other forms of pentastomiasis which are typically asymptomatic, occur much more frequently. Any potential health consequences of asymptomatic pentastomiasis remain entirely unknown. Ocular pentastomiasis may thus be regarded as a sentinel form for pentastomiasis in general, for it is easily detectable.
- Ocular adnexal localization of pentastomid nymphs has a good prognosis after surgical intervention, while intraocular parasites usually cause permanent visual damage.

**Top Five Papers**

- Tappe D, Büttner DW. (2009) Diagnosis of human visceral pentastomiasis. PLoS Negl Trop Dis. 5:e320.
- Tappe D, Meyer M, Oesterlein A, Jaye A, Frosch M, et al. (2011) Transmission of *Armillifer armillatus* ova at snake farm, The Gambia, West Africa. Emerg Infect Dis 17: 251–254.
- Koehsler M, Walochnik J, Georgopoulos M, Pruente C, Boeckeler W, et al. (2011) *Linguatula serrata* tongue worm in human eye, Austria. Emerg Infect Dis. 17(5): 870–872
- Hardi R; Sulyok M; Rozsa L, Bodo I. (2013) A man with unilateral ocular pain and blindness Clin Inf Dis 57: 469–470.
- Lazo RF, Hidalgo E, Lazo JE, Bermeo A, Llaguno M, et al. (1999) Ocular linguatuliasis in Ecuador: Case report and morphometric study of the larva of *Linguatula serrata*. Am J Trop Med Hyg 60: 405–409.

## Supporting Information

Video S1 *A. grandis* nymph in the left eye, Sankuru district, Democratic Republic of the Congo, case 1. An annulated foreign body was identified in the anterior chamber showing peristaltic motion. The eye was clipped under retrobulbar anesthesia, and the cornea was incised at the limbus, then the parasite was extracted from the anterior chamber.
(MP4)
